# Effects of different medial arch support heights on rearfoot kinematics

**DOI:** 10.1371/journal.pone.0172334

**Published:** 2017-03-03

**Authors:** Gunnar Wahmkow, Michael Cassel, Frank Mayer, Heiner Baur

**Affiliations:** 1 University outpatient clinic, University of Potsdam, Department of Sports Medicine, Am Neuen Palais 10, Haus 12, Potsdam, Germany; 2 Bern University of Applied Sciences, Health, Physiotherapy, Murtenstrasse 10, Bern, Switzerland; Tokai University, JAPAN

## Abstract

**Background:**

Foot orthoses are usually assumed to be effective by optimizing mechanically dynamic rearfoot configuration. However, the effect from a foot orthosis on kinematics that has been demonstrated scientifically has only been marginal. The aim of this study was to examine the effect of different heights in medial arch-supported foot orthoses on rear foot motion during gait.

**Methods:**

Nineteen asymptomatic runners (36±11years, 180±5cm, 79±10kg; 41±22km/week) participated in the study. Trials were recorded at 3.1 mph (5 km/h) on a treadmill. Athletes walked barefoot and with 4 different not customized medial arch-supported foot orthoses of various arch heights (N:0 mm, M:30 mm, H:35 mm, E:40mm). Six infrared cameras and the `Oxford Foot Model´ were used to capture motion. The average stride in each condition was calculated from 50 gait cycles per condition. Eversion excursion and internal tibia rotation were analyzed. Descriptive statistics included calculating the mean ± SD and 95% CIs. Group differences by condition were analyzed by one factor (foot orthoses) repeated measures ANOVA (α = 0.05).

**Results:**

Eversion excursion revealed the lowest values for N and highest for H (B:4.6°±2.2°; 95% CI [3.1;6.2]/N:4.0°±1.7°; [2.9;5.2]/M:5.2°±2.6°; [3.6;6.8]/H:6.2°±3.3°; [4.0;8.5]/E:5.1°±3.5°; [2.8;7.5]) (*p>0*.*05*). Range of internal tibia rotation was lowest with orthosis H and highest with E (B:13.3°±3.2°; 95% CI [11.0;15.6]/N:14.5°±7.2°; [9.2;19.6]/M:13.8°±5.0°; [10.8;16.8]/H:12.3°±4.3°; [9.0;15.6]/E:14.9°±5.0°; [11.5;18.3]) (*p>0*.*05*). Differences between conditions were small and the intrasubject variation high.

**Conclusion:**

Our results indicate that different arch support heights have no systematic effect on eversion excursion or the range of internal tibia rotation and therefore might not exert a crucial influence on rear foot alignment during gait.

## Background

The prevalence of running-related injuries has risen recently due to the growing number of recreational runners [1]. Numerous gait analysis investigations have focused on the clinical relevance of excessive pronation during walking or running, as it has been assumed to be a major factor in the development of lower extremity overuse injuries or complaints of anterior knee pain [2,3], tendinopathy and arthritis symptoms [4]. However, scientific evidence for a relation between skeletal alignment and the prevalence of running-related overuse injuries through excessive pronation has been demonstrated in neither marathon runners nor triathletes [5,6], nor in novice runners[7].

The static anatomical structure of the medial arch is believed to play a key role in the human gait’s dynamic function [8]. The natural mechanism is lowering of the medial arch to absorb impact during the stance phase while walking or running. This mechanism is also known as “foot pronation”, which can be divided into rearfoot eversion as the main factor, which is accompanied by the forefoot’s abduction and dorsiflexion [1].

Still assuming that abnormal pronation leads to complaints, the shoe industry and orthotic designers have attempted to alter poor statics with the goal of preventing injuries through improved dynamic function. An orthotic design entailing semi-rigid support should restrict an abnormal range of motion (ROM) [4]. However, kinematic analysis has revealed inconsistent findings concerning the paradigm of skeletal alignment through orthotics via “antipronatory features”. Some researchers detected less rearfoot peak eversion by using medially-supported orthoses in asymptomatic runners [9,10] and in those with anterior knee pain [11]. Moreover, the use of medially-wedged orthoses was reported to reduce the initial eversion angle [12] as well as peak eversion and eversion excursion [11]. Identical effects were observed [13,14] with posted and molded orthoses. Furthermore, less internal tibia rotation was described using semi-rigid, custom-moulded orthoses [15] or orthoses supported medial- anteriorly and posteriorly [16]. In contrast, other studies showed no effect on peak eversion, eversion excursion [17,18] or on internal tibia rotation with a 4° medially-wedged orthosis [11] or 7° varus wedge [19]. This controversy is also addressed in a meta-analysis by Mills et al. [20], which revealed only marginal effects on kinematics by the use of foot orthoses.

Nevertheless, foot orthoses are widely used in clinical practice to limit abnormal rear foot pronation angles [4,21]. In particular, the use of medial arch support seems to be clinically the first choice when treating excessive rearfoot pronation. The aim of this study was therefore to investigate the effect of different heights of medial arch support in foot orthoses on kinematic rearfoot parameters during gait.

## Methods

### Subjects

Nineteen healthy male subjects between 18 and 60 years of age were included in this study. All subjects signed a written informed consent, and were all orthopedically examined by the same physician (MC) for normal foot flexibility, ankle ROMS, normal arch height and the absence of any foot pathologies or deformities. The study was approved by the local ethics committee of Potsdam University (no number given, but letter of approval is attached).

Subjects´ anthropometric and training data are presented in Table 1. Inclusion criteria were a minimum of three training sessions per week as a leisure or competitive runner and the absence of lower extremity injuries or complaints within the last six months. We assumed that a cohort of runners would exhibit a stabler gait pattern than inexperienced subjects to reduce intraindividual variability during measurements [22,23]. Anamnestically documented operations on the lower extremities led to study exclusion.

**Table 1:** Subjects`anthropometric data, training history and weekly running mileage.

Parameter	Range	Mean±SD
n		19
Age in [Y]	22–57	36 ± 11
Weight in [kg]	57–97	79 ± 10
Height in [cm]	170–190	180 ± 5
Years of training [J]	1,5–30	12 ± 7
Running mileage [km/w]	12–90	41 ± 22

### Materials

**Fig 1:**
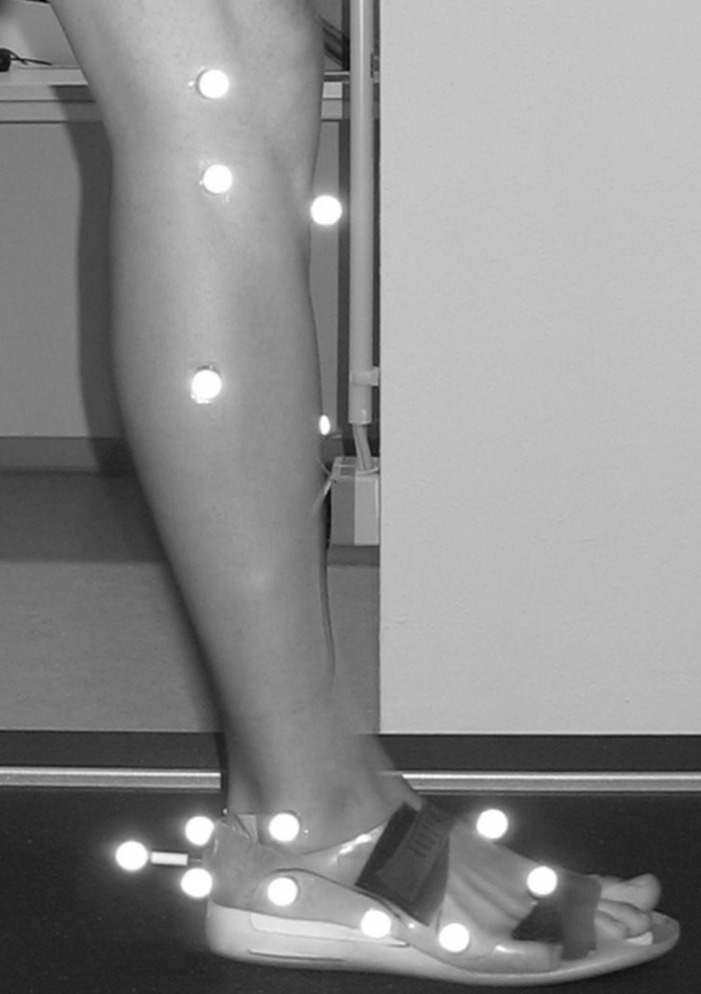
Subjects’ lower extremity and foot in customised silicon slippers prepared with skin-based markers following the `Oxford Foot Model´ guidelines.

**Fig 2:**
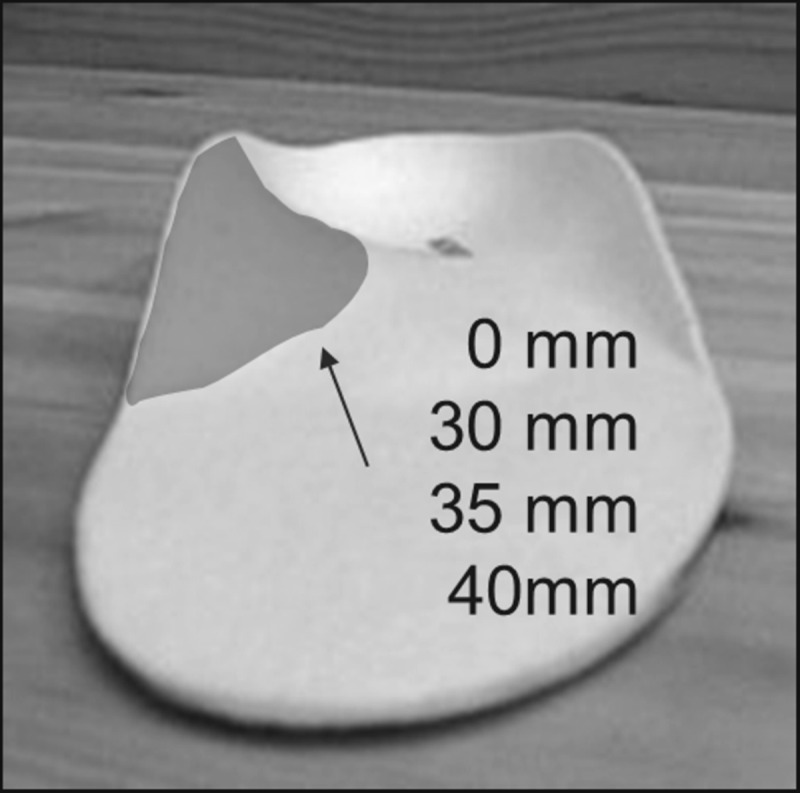
Orthosis with medial arch support (grey area) in different heights.

Six infrared cameras (Vicon MX 3, Vicon Motion Analysis Ltd., Oxford, UK) with a sampling frequency of 200 Hz were used to capture motion. Marker setup preparation consisted of 28 reflective skin markers (Ø 14 mm) following the `Oxford Foot Model´ guidelines [24–26]. Seventeen markers were placed on the lower right leg, 13 of them on the right foot (Fig 1). Furthermore, to eliminate shoe effects, each subject had to wear standardized silicon slippers (custom-made for study purpose, not commercially available, shown in Fig 1, IETEC®, Künzell, Germany) combined with an insole (condition: N, H, M, E) or without an insole (barefoot condition). Those slippers had holes cut in them to avoid influencing skin markers and to keep them attached to the subjects’ foot without requiring replacement after changing orthosis conditions (refer to [11]). After preparing and getting accustomed to treadmill walking (Cybex Legacy 750T, Medway, U.S.A) all subjects had to walk in each condition in randomized order (*www.randomazation.com*) for one minute at a given speed of 3.1 mph (5 km/h). This speed was chosen as a fast walking speed instead of running to minimize intraindividual variability between gait cycles. Different sizes of identical, not customized foot orthoses (MoveControl®, IETEC® GmbH, Fulda, Fig 2) made of polyurethane foam material (shore 25; with an ethylene vinyl acetate EVA core, shore 55, compression moulded and semirigid) with a concave-shaped heel and medial arch support in different heights (Table 2) were used to fit all subjects [27].

**Table 2:** condition, medial arch support heights.

Condition	Height of medial arch [mm]
B	barefoot
N	0 mm
M	30 mm
H	35 mm
E	40 mm

### Data processing

Kinematic raw data were processed with Vicon Nexus 1.4.115 software (Vicon Motion Systems Ltd., Oxford, UK). Data from one static trial per subject was reconstructed and markers automatically or manually labelled and then used as reference for further data processing. For the dynamic trials, gaps in marker trajectories were filled with pattern fill (calculated trajectory) or spline fill (trajectories from similar markers) and checked for plausibility (the maximum gap fill used was 5 frames). To smooth the trajectories, we applied a Woltering filter. Gait cycle events (heel strike, toe off) were then manually identified by tracking the lowest point of heel marker trajectories at initial touch down and the lowest point of toe marker trajectories just before toe off. Using the Vicon Workstation Software (Vicon Motion Systems Ltd., Oxford, UK) all 28 marker positions were calculated and used to obtain segment angles of the pelvis and lower limb. Segments of particular interest were the tibia, rearfoot, and forefoot. As pronation is the sum of movements between foot segments based on rearfoot eversion, abduction and dorsiflexion, it is necessary to create separate variables of interest. Rearfoot eversion angles have usually been used in the literature to represent pronation during walking or running. The `Oxford Foot Model´ allows angle calculations between the segments of rearfoot, forefoot, and tibia in terms of each other and the floor. Angle values for rotations in the `Oxford Foot Model´ are defined to appear anticlockwise in the Z-plane and clockwise in the X-plane (for more details on the Oxford Foot Model, please see references 24–26). After normalization to the static trial variables were calculated from the initial angle at heel strike (defined as the minimum angle) subtracted from the maximum-attained angles during the stance phase in the plane where they were expected to occur in a functional manner (Z and X). All variables can be understood as an excursion (ROM) over time.

### Outcome variables

Three variables of interest were determined from the differences in the absolute angle values measured at the time point of initial heel contact and in the maximum angle (Table 3). We calculated two variables of interest in the frontal plane to determine rearfoot eversion (Z): 1) rearfoot to tibia (right hindfoot to tibia in Z) and 2) rearfoot to floor (right hindfoot to floor in Z). To measure the internal tibia rotation (considered a main reason for lower extremity complaints in the literature [3,15,28,29]) the third variable was calculated in the horizontal plane (X): 3) internal tibia rotation (right hindfoot to tibia in X). For detailed information on output angles and calculated angles see S1 Table.

**Table 3:** Absolute values for eversion angles and tibial internal rotation at the point of heel strike and maximum (heel strike to maximum [°]) for different foot orthosis conditions.

Event	Condition	Eversion±SD		Internal Tibia Rotation±SD
		Rearfoot to Tibia(Z)	Rearfoot to floor(Z)	Rearfoot to Tibia (X)
**Heel Strike**	B	-0.9±5.4	3.0±6.5	6.4±4.6
	N	-3.6±6.0	-1.3±6.9	6.8±4.4
	M	-3.3±6.7	0.9±7.6	8.2±7.3
	H	-2.4±6.0	-2.7±12.4	7.4±6.0
	E	-1.5±8.5	1.0±7.2	7.2±7.0
**Maximum**	B	-10.2±5.9	-1.6±6.8	-6.9±4.7
	N	-12.6±6.2	-5.3±6.8	-7.6±9.1
	M	-13.4±6.9	-4.3±6.3	-5.7±8.8
	H	-12.7±6.0	-8.9±11.7	-4.9±5.2
	E	-12.2±7.8	-4.1±6.9	-7.7±8.1

### Statistical analysis

After excluding any implausible data, missing markers or incomplete trials, excursions and ranges were finally calculated for 14 subjects by using JMP5.0.1, statistical discovery software (SAS Institute Inc.). Descriptive statistical analysis for outcome variables was made by calculating the mean ± standard deviation, standard error of the mean and 95% confidence intervals based on the averages of 50 right-footed stance phases out of 50 gait cycles per subject per condition. Group differences by condition were analyzed by one factor (foot orthoses) repeated measures ANOVA (α = 0.05).

## Results

Mean angle values according to footwear condition at heel strike and maximum are displayed in Table 3.

### Eversion excursion

The mean eversion excursion angles for the different footwear conditions, standard deviation, 95% confidence intervals as well as standard error of the mean are illustrated in Table 4. The rearfoot to tibia and rearfoot to floor excursion from initial heel strike to maximum value ranged from 9.0°±2.2° (N) to 10.7°±4.0° (E) and 4.0°±1.7° (N) to 6.2°±3.3° (H), respectively. We observed no statistically significant differences among the various conditions (*p>0*.*05*).

**Table 4:** Eversion excursion angles and internal tibia rotation (Δ heel strike to maximum [°]) for different foot orthosis conditions.

Movement	Condition	Mean ±SD	±95% CI	Std Error Mean
Eversion excursion				
Rearfoot to tibia (Z)	B	9.3±2.2	7.7;10.9	0.7
	N	9.0±2.2	7.5;10.5	0.7
	M	10.1±3.5	8.0;12.3	1.0
	H	10.2±3.6	7.8;12.7	1.1
	E	10.7±4.0	8.0;13.4	1.2
Rearfoot to floor (Z)	B	4.6±2.2	3.1;6.2	0.7
	N	4.0±1.7	2.9;5.2	0.5
	M	5.2±2.6	3.6;6.8	0.7
	H	6.2±3.3	4.0;8.5	1.0
	E	5.1±3.5	2.8;7.5	1.0
Internal tibia rotation				
Rearfoot to tibia(X)	B	13.3±3.2	11.0;15.6	1.0
	N	14.5±7.2	9.2;19.6	2.3
	M	13.8±5.0	10.8;16.8	1.4
	H	12.3±4.3	9.0;15.6	1.4
	E	14.9±5.0	11.5;18.3	1.5

### Internal tibia rotation

For internal tibia rotation the H condition presented the lowest mean values (12.3°±4.3°) while the insole E condition presented the highest mean value (14.9°±5.0°, Table 4). There were no statistically significant group differences (*p>0*.*05*).

## Discussion

The aim of this study was to examine the effect of different medial arch support heights in foot orthoses on kinematic parameters during gait. We detected no statistically significant differences in rearfoot eversion excursions or ranges of internal tibia rotation among conditions.

This finding is in line with results from Stacoff et al. [16,17]. Their study revealed no statistically significant changes in total calcaneal eversion by orthoses with medial arch support using calcaneal bone pins in five subjects calculating means of three to five individual trials per condition. They stated that those findings were not systematic across subjects. Moreover, only small, nonsystematic differences between conditions were observed. They also showed that the variability among subjects is greater than among conditions. In accordance to Stacoff’s findings [16,17], the present study revealed very high intraindividual and interindividual variability. Our methodological approach to reduce variability was firstly to choose a mostly homogeneous group of runners, where it was believed that individual gait patterns might be more stable than in nonrunners [22,23]. Secondly, we calculated an average of 50 strides per subject per condition. This will remain important in the future, especially when small differences are expected. This may account for the natural variability in intrasubject gait patterns. Furthermore, the `Oxford Foot Model´ was used to obtain detailed information from the rear foot segment and tibia. Compared to older studies at the beginning of 3D kinematic measurements, today computational capacity, data storage and processing expenses in general are not important factors anymore. Therefore, averages out of numerous strides from complex foot models like the used `Oxford Foot Model´ can be calculated easily. After all, variability between subjects in the response to measured insole conditions point towards individual responses to foot orthoses with no clear trend or direction for the measured cohort.

Data from the present study revealed larger rearfoot excursions when wearing orthoses with longitudinal arch support. The smallest excursions for rearfoot eversion as well as ranges of internal tibia rotation were found in barefoot walking (B) and neutral condition (N) compared to all other insole conditions. Instead of reducing the ROM, a thin arch support seems to be able to enlarge the natural joint motion during gait. This fact contradicts the `paradigm of skeletal alignment´ as well as the results presented by Liu et al. [12] and Rodriguez et al. [11]. Although our results exhibited no statistical significance due to broad variation in the outcome variables, we observed this effect in each subject. This observation is also supported by earlier findings from Donoghue et al. [30] in their 12 subjects with chronic Achilles tendinopathy. There, a larger peak eversion and eversion excursion in conjunction with shod (their own running shoes plus customized orthoses) running than with barefoot running was reported. Likewise, Williams et al. [18] detected no effect in 11 runners on eversion excursion but larger ranges of tibia rotation when using varus wedges ranging from 5° to 25°.

In their meta-analysis, Mills et al. [20] pooled data that revealed no reduction in eversion excursion, and a slight reduction in the internal tibia rotation of around 1,5°. They thus demonstrated only marginal effects from different types of orthoses onto eversion excursion and internal tibia rotation, indicating that current standard skin-based 3D kinematic analysis is probably unable to detect the very small magnitude of changes in mechanical rearfoot bone configuration during walking.

While most previous studies either compared orthoses versus a control condition or different orthotic designs to each other, this is the first study to have analyzed the effect of orthoses differing only in arch support heights on rearfoot kinematics. Contrary findings in the literature reveal that clarifying the effect of anti-pronation devices taking the purely mechanical approach is limited. The current study delivers further evidence of the need to reconsider the mechanical approach’s utility in explaining the clinically-demonstrated positive effects of orthoses [31,32]. The kinematic effects of orthoses with medial arch support are probably smaller than we have assumed so far, and are therefore unsuitable on their own for explaining the clinical effects of insoles.

Nevertheless, clinical trials have shown that foot orthoses made of similar polyurethane foam materials play a decisive role in the treatment of running-related injuries. Custom-made, semi-rigid running shoe insoles have been proven to have evident effects on pain relief and improvement in function in runners with overuse injuries of the lower extremity [31,32,33]. From a clinical perspective, it is therefore indicated to prescribe insoles for symptom reduction. However, the mechanism behind this clinical benefit of insoles is still unclear. Results of the present study show that well-known and usually used kinematic quantities (eversion excursion/tibia internal rotation) are limited in differentiating between various medial arch support heights used in healthy subjects. Therefore, we assume that the examined kinematic quantities are probably not capable to explain reported clinical effects.

Some investigators propose neuromuscular effects of insoles [34,35]. Nigg et al. [35,36] developed a sensorimotor theory in which forces underneath the foot sole are modified by foot orthoses. Those forces act as input signals to muscles and other origins of proprioception. This modified afferent input may affect motor output. Consequently, gait patterns should remain the same with almost no kinematic changes but involving altered muscle action. Moreover, another randomized controlled trial in 99 runners with overuse injury reported that foot orthoses did enhance peroneal preactivation before heel-strike. This was interpreted as a change in motor program leading to better ankle stability. Eventually, this might point to a modified afferent input on the foot sole (or other proprioceptive structures), which leads to a change in muscular control [37]. This descriptive observation does not directly lead to a new `sensorimotor´ paradigm but it opens the field for new methodological approaches like the integration of neurophysiologic techniques like h-reflex measurements or transcranial magnetic stimulation (TMS) [38]. Data supporting those paradigms is sparse but there seems to be potential in new neuromechanic or sensorimotor approaches.

### Limitations

Several limits of our study design should be considered. Kinematic data was collected with skin-based markers. This is standard in clinical gait analyses and a useful tool for evaluating gait patterns. However, this method can only approximate genuine structural movement due to skin moving over the bony landmarks during locomotion. Moreover, whether those expected small differences are clinically relevant is debatable. Another limitation is the gait velocity chosen during measurements. When evaluating gait kinematics, one can expect larger dynamic ranges in joint movements at higher velocities, as well as fatigue [39]. Extrapolating the current results to running can therefore only be done with caution. By considering the heel strike as the minimum eversion angle for further excursion calculations, typical gait patterns [1] were noted. If there were any smaller angles between heel off and toe off is theoretically not assumed but not known for sure.

## Conclusion

Foot orthoses do not seem to have a crucial influence on typical rearfoot kinematic outcomes as do rearfoot eversion or internal tibia rotation during walking gait. We observed high intersubject variability in motion independent of the height of medial arch support. Kinematic analysis might therefore not be the sole method of choice with which to detect the biomechanical effects of foot orthoses. There may be other mechanisms involved (i.e., sensorimotor regulation theories). Further investigation of those effects will require the development of new methodological paradigms to deepen the knowledge of the effects of foot orthoses on movement.

## Supporting information

S1 Table (XLSX)
